# Prospective Evaluation of Cytology, CINtec^®^ and PD-L1 for the Detection of Cervical Intraepithelial Neoplasia: A Single-Center Study

**DOI:** 10.3390/jcm15031171

**Published:** 2026-02-02

**Authors:** Alexandru Hamod, Mihaela Grigore, Ingrid-Andrada Vasilache, Ramona-Gabriela Ursu, Oancea Mihaela, Razvan Popovici, Ana-Maria Grigore, Ludmila Lozneanu, Dan-Constantin Andronic, Manuela Ciocoiu

**Affiliations:** 1 Grigore T. Popa University of Medicine and Pharmacy, 700115 Iasi, Romania; alexandru.hamod@umfiasi.ro (A.H.);; 2 IInd Department of Obstetrics Gynecology, Iuliu Haţieganu University of Medicine and Pharmacy Cluj-Napoca, 13 Emil Isac, 400023 Cluj-Napoca, Romania

**Keywords:** cervical intraepithelial neoplasia, screening, cytology, CINtec^®^, PD-L1

## Abstract

**Background/Objectives**: This study evaluated the diagnostic accuracy of cervical cytology, CINtec^®^ (p16/Ki-67 dual staining), and PD-L1 immunohistochemistry, individually and in combination with high-risk HPV (HR-HPV) testing, for identifying histologically confirmed cervical lesions ranging from CIN1 to invasive carcinoma. **Methods**: We conducted a prospective cross-sectional study including 114 patients who underwent cervical cytology, CINtec^®^, PD-L1 staining, HPV genotyping, and histopathologic confirmation at a tertiary clinical center between September 2024 and September 2025. Sensitivity, specificity, PPV, NPV, and ROC performance were calculated for each test across lesion categories. Multivariable logistic regression models incorporating HR-HPV status were used to assess added predictive value. **Results**: All tests showed poor performance for CIN1 (cytology AUC 0.488; CINtec^®^ 0.374; PD-L1 0.366). Diagnostic accuracy improved markedly with lesion severity. For CIN3, CINtec^®^ demonstrated the highest discriminative ability (AUC 0.826), with cytology and PD-L1 also performing well (AUC 0.820 and 0.753). Cytology achieved the strongest ROC performance for CIN2+ (AUC 0.937), CIN3+ (0.913), and invasive carcinoma (0.887). PD-L1 consistently showed lower accuracy across categories. Cytology + HR-HPV demonstrated the highest AUC across all lesion categories. **Conclusions**: Cytology and CINtec^®^ exhibited strong diagnostic accuracy for high-grade lesions, while PD-L1 showed limited utility as an independent screening marker. Combining cytology with HR-HPV testing enhanced predictive performance across all lesion categories. These findings support the continued use of cytology-based triage and highlight CINtec^®^ as a valuable adjunct for high-grade disease detection. Because this study used a high-prevalence referral cohort, specificity may be overestimated and not representative of population-based screening.

## 1. Introduction

Cervical intraepithelial neoplasia (CIN) represents a spectrum of premalignant changes in the cervical epithelium, primarily driven by persistent high-risk human papillomavirus (HPV) infection. Early and accurate detection of CIN is critical for preventing progression to cervical cancer, a leading cause of cancer-related morbidity and mortality among women worldwide. While cytology is widely used and accessible, its sensitivity for detecting high-grade lesions (CIN2+) is limited, with reported sensitivities ranging from 55% to 76.6% and specificities from 49.6% to 95.4%, depending on the population and threshold used [1,2].

To address these limitations, novel biomarkers and immunocytochemical assays have been developed. Among these, the p16/Ki-67 dual stain (CINtec^®^) appeared to be a useful complementary screening tool [3,4]. p16 is a cell cycle regulatory protein that is overexpressed in response to oncogenic HPV infection, while Ki-67 is a marker of cellular proliferation. Their co-expression in the same cell is abnormal and indicates cell cycle deregulation due to transforming HPV infection, which is strongly associated with high-grade cervical lesions [5,6]. Studies showed that p16/Ki-67 dual staining achieved sensitivities for CIN2+ detection between 76.9% and 97.3% and specificities between 58.9% and 99.3%, often outperforming cytology and providing higher specificity than HPV testing alone [1,2,7,8,9,10,11]. For example, a large prospective study found dual-stained cytology had a sensitivity of 86.7% and specificity of 95.2% for CIN2+, compared to 68.5% and 95.4% for cytology [1]. In HPV-positive women, dual staining maintained high sensitivity (91.2% for CIN2+) and improved specificity over HPV genotyping or cytology alone [9,11].

PD-L1 is an immune checkpoint protein whose expression is upregulated in cervical dysplasia and cancer, reflecting immune evasion by neoplastic cells [12]. PD-L1 positivity in cervical cancer varies widely by study and detection method, with reported rates from 34% to over 90% in squamous cell carcinoma and 16–60% in adenocarcinoma [13,14,15]. However, data on PD-L1 diagnostic accuracy for cervical dysplasia are lacking in the current literature, and its clinical utility remains to be established. In this study, PD-L1 expression was evaluated exploratorily as a marker of biological progression and immune escape, rather than as a candidate screening or triage biomarker.

Despite the promising performance of p16/Ki-67 dual staining and the theoretical advantages of PD-L1, there is ongoing controversy and insufficient prospective validation regarding their optimal use in routine screening and triage. Furthermore, the performance of these markers in self-collected samples, diverse age groups, and resource-limited settings is not well established.

The aim of this study was to test the diagnostic accuracy of cervical cytology, CINtec^®^ and PD-L1 for the detection of cervical dysplasia, considering cervical biopsy results as the gold standard, in a cohort of patients recruited from a single center in Romania.

## 2. Materials and Methods

We conducted a prospective cross-sectional study that included referred patients who underwent both cervical screening and histopathologic evaluation at Cuza voda Clinical Hospital of Obstetrics and Gynecology, Iasi, Romania, between September 2024 and September 2025. Additional screening tests (CINtec^®-^ CINtec^®^ PLUS Cytology kit, Roche mtm Laboratories AG, Mannheim, Germany, PD-L1 immunohistochemistry) for cervical dysplasia were performed. We included patients with available results for cytology, CINtec^®^ (p16/Ki-67 dual immunostaining), PD-L1 immunohistochemistry, and HPV genotyping, as well as a corresponding cervical biopsy, who gave their informed consent for participation in the study.

Patients were excluded from the study if any of the following applied: absence of a histopathologic diagnosis, incomplete screening test results, prior treatment for cervical intraepithelial neoplasia or cervical cancer, or insufficient clinical data for the variables of interest (age, body mass index, reproductive and behavioral history). The final analytical sample comprised 114 patients.

The study was conducted in accordance with the Declaration of Helsinki. The protocol was approved by the local institutional ethics committees (Cuza voda Clinical Hospital of Obstetrics and Gynecology-11630/06.09.2024; Grigore T. Popa University of Medicine and Pharmacy Iasi-480/21.10.2024), and written informed consent was obtained from all participants prior to enrollment.

Cervical biopsies were processed according to standard pathology protocols and reviewed by experienced gynecologic pathologists who were blinded to the screening test results. Histologic outcomes were classified into five categories: cervical intraepithelial neoplasia grade 1 (CIN1), CIN2, CIN3, invasive carcinoma, and negative histology (no intraepithelial lesion or malignancy).

For diagnostic accuracy analyses, additional composite endpoints were defined as follows:

- CIN2+: presence of CIN2, CIN3, or invasive carcinoma;
- CIN3+: presence of CIN3 or invasive carcinoma.

These histopathologic categorizations served as the reference standard for all test performance and ROC analyses.

Conventional or liquid-based cervical cytology was performed according to routine clinical practice. Cytologic results were reported using standard terminology and categorized as:

- NILM (negative for intraepithelial lesion or malignancy),
- LSIL (low-grade squamous intraepithelial lesion),
- HSIL (high-grade squamous intraepithelial lesion),
- ASC-US (atypical squamous cells of undetermined significance), and
- ASC-H (atypical squamous cells, cannot exclude HSIL).

For the purposes of diagnostic performance analysis, cytology was dichotomized as positive (ASC-US, ASC-H, LSIL, or HSIL) or negative (NILM).

CINtec^®^ p16/Ki-67 dual-stain testing was performed on cervical specimens according to the manufacturer’s instructions (CINtec^®^ PLUS Cytology kit, Roche mtm Laboratories AG, Mannheim, Germany). CINtec^®^ results were reported qualitatively and coded as positive or negative based on the presence of at least one dual-stained epithelial cell.

PD-L1 expression was assessed by immunohistochemistry (IHC) on formalin-fixed, paraffin-embedded tissue using a standardized protocol. Tissue blocks were sectioned at 4 μm, deparaffinized in xylene, and rehydrated through graded ethanol. Endogenous peroxidase activity was blocked by incubation with 3% hydrogen peroxide for 10 min. Heat-induced antigen retrieval was performed using citrate buffer (pH 6.0).

Sections were incubated with a mouse monoclonal anti-PD-L1 antibody (clone CAL20; Abcam, Cambridge, MA, USA) at a dilution of 1:500, followed by detection using the streptavidin–biotin–peroxidase complex method and diaminobenzidine as chromogen. Human tonsillar tissue was included as a positive control in each staining run, and omission of the primary antibody served as a negative control.

PD-L1 expression was evaluated exclusively in dysplastic squamous epithelial cells. Only membranous staining (partial or complete) of viable dysplastic cells was considered specific and included in scoring; cytoplasmic staining was recorded but not considered for determination of positivity. Staining intensity was not used as a criterion for positivity.

PD-L1 expression was assessed using the Tumor Proportion Score (TPS), defined as the percentage of PD-L1–positive dysplastic epithelial cells relative to the total number of viable dysplastic cells: number of PD-L1–positive tumor cells ÷ total number of viable tumor cells) × 100.

A cut-off value of ≥1% membranous staining was used to define PD-L1 positivity, while cases with <1% staining were classified as negative. This cutoff was selected based on previously published studies in cervical intraepithelial neoplasia and related squamous lesions. The Combined Positive Score (CPS) was not applied, as it is not currently validated or routinely used for PD-L1 evaluation in cervical dysplasia.

All slides were independently evaluated by two experienced pathologists who were blinded to clinical and histopathological data. In cases of discrepant interpretation regarding PD-L1 positivity, a consensus score was reached following joint review.

HPV genotyping was performed on cervical samples using a validated molecular assay, Allplex™ HPV28 Detection (Seegene Technologies Inc Europe, Dusseldorf, Germany), capable of differentiating high-risk (HR-HPV) and low-risk (LR-HPV) genotypes. For analysis, HR-HPV positivity, LR-HPV positivity, and HPV coinfection (simultaneous detection of at least one high-risk and one low-risk genotype) were coded as binary variables.

Clinical and demographic data were collected from medical records and structured clinical forms. The following variables were included: age (years), body mass index (BMI, kg/m^2^), number of pregnancies, place of residence, history of HPV infection, history of sexually transmitted infections (STIs), smoking, alcohol consumption, hormonal contraceptive use, immunosuppression, HPV vaccination status, and HR-HPV positivity.

All analyses were performed using Stata (version 19.5, StataCorp LLC, College Station, TX, USA). A two-sided *p*-value < 0.05 was considered statistically significant. Continuous variables were summarized as means and standard deviations (SD). Differences across the five histopathologic groups (CIN1, CIN2, CIN3, invasive carcinoma, and negative histology) were assessed using one-way analysis of variance (ANOVA) and Bonferroni post hoc tests. These results are presented in Table 1, with detailed pairwise comparisons provided in Supplementary Tables S1–S3.

Categorical variables were summarized as counts and percentages within each histopathologic category. Associations between each categorical variable and biopsy group were evaluated using the χ^2^ test. When expected cell counts were <5 in any cell of the contingency table, Fisher’s exact test was applied instead.

For each histologic definition of disease (CIN1, CIN2, CIN3, CIN2+, CIN3+, and invasive carcinoma), cytology, CINtec^®^, and PD-L1 were evaluated as index tests. Using 2 × 2 contingency tables, we calculated sensitivity, specificity, positive predictive value (PPV), and negative predictive value (NPV), each with corresponding 95% confidence intervals (CIs). Receiver operating characteristic (ROC) curves were constructed for each test and lesion category, and the area under the ROC curve (AUC) with 95% CI was estimated using a non-parametric approach.

For each histological endpoint (CIN1, CIN2, CIN3, invasive carcinoma), a multivariable logistic regression model was constructed with cytology, CINtec^®^, and PD-L1 test results as predictors. All predictors were coded as binary variables (0 = negative, 1 = positive). The outcome variable was the presence or absence of the respective histological category. Predicted probabilities from each model were used to generate ROC curves along with 95% CI. We also reported the β coefficients derived from the multivariable logistic regression model

To evaluate the incremental value of HPV genotyping when combined with different screening tests, we constructed multivariable logistic regression models using the presence versus absence of each lesion category as the binary outcome. For each model, predicted probabilities were used to generate ROC curves and to estimate the area under the AUC with 95% confidence intervals.

For each lesion definition, three separate models were fitted, each incorporating HR-HPV positivity together with one screening marker:

- Model 1: cytology plus HR-HPV positivity;
- Model 2: CINtec^®^ positivity plus HR-HPV positivity;
- Model 3: PD-L1 positivity plus HR-HPV positivity.

## 3. Results

Table 1 comprises the ANOVA analysis results from the comparison of continuous variables. The mean age was significantly higher among patients with invasive carcinoma compared to patients diagnosed with CIN1 (mean and standard deviation: 34.65 ± 9.37 years, *p* = 0.20). Patients diagnosed with CIN2 (mean and standard deviation: 37.07 ± 9.42 years, *p* = 0.378) and CIN3 (mean and standard deviation: 36.14 ± 8.75 years, *p* = 0.122) were also younger than patients with invasive carcinoma, but the differences were not statistically significant (Table S1. Age comparison between groups (Bonferroni)).

Body mass index also demonstrated significant variation between groups (*p* = 0.0218). Patients with invasive carcinoma exhibited the highest mean BMI (27.24 ± 3.72 kg/m^2^), whereas those with CIN2 and CIN3 had the lowest values (23.29 ± 3.76 and 22.84 ± 4.11 kg/m^2^, respectively). The mean BMI of patients with invasive carcinoma was significantly higher than that of patients diagnosed with CIN3 → (*p* = 0.025)—Table S2. BMI comparison between groups (Bonferroni). Patients diagnosed with CIN1 showed an intermediate BMI profile (25.18 ± 3.79 kg/m^2^).

The number of pregnancies did not vary significantly among biopsy groups (*p* = 0.167). Although the patients diagnosed with invasive carcinoma had a numerically higher mean number of pregnancies (1.58 ± 1.56), pregnancy counts in the CIN1, CIN2 and CIN3 patients were similar, and no statistically meaningful differences were detected—Table S3. Number of pregnancy comparisons between groups (Bonferroni).

Table 2 comprises the baseline clinical and demographic characteristics according to histopathologic diagnosis. A history of HPV infection was significantly associated with histopathologic category (*p* < 0.0001), with the highest proportions observed in the CIN1 (34.1%), CIN3 (23.1%), and CIN2 (15.4%) groups. A history of sexually transmitted infections also varied significantly across groups (*p* = 0.006), with the CIN2 group accounting for 75% of all STI-positive cases. Smoking history demonstrated a significant distributional imbalance as well (*p* = 0.012), with the greatest proportion of smokers in CIN3 (33.3%), followed by CIN2 (23.3%) and invasive carcinoma (16.7%).

HPV vaccination status differed substantially between groups (*p* = 0.028), with CIN1 demonstrating the highest proportion of vaccinated participants (40.6%), followed by CIN3 (28.1%) and negative cases (21.9%), while no vaccinated individuals were identified in the invasive carcinoma group. High-risk HPV positivity also varied markedly (*p* < 0.0001), with the highest proportions in CIN1 (30.1%), CIN3 (23.7%), and CIN2 (16.1%). Low-risk HPV positivity was strongly associated with CIN1 (*p* = 0.002).

Cytologic findings showed the expected strong alignment with histopathologic outcomes. ASC-H occurred exclusively in the CIN3 group (*p* < 0.0001), HSIL was most prevalent in CIN3 (45.7%) and invasive carcinoma (34.3%), LSIL was primarily associated with CIN1 (73.8%) and CIN2 (19.0%), and NILM was observed exclusively among individuals with negative biopsy results (100%). CINtec^®^ positivity demonstrated significant variation across groups (*p* < 0.0001), with the highest prevalence in CIN3 (40.7%), followed by invasive carcinoma (22.2%) and CIN2 (20.4%). PD-L1 positivity was similarly associated with more advanced histopathology (*p* < 0.0001), with CIN3 showing the highest proportion (53.9%), followed by invasive carcinoma (30.8%).

Table 3 comprised the diagnostic performance of individual tests for the detection of cervical lesions across histopathological categories. These findings should be interpreted with caution, as the referral-based study design may overestimate specificity results. For CIN1, all three tests demonstrated poor diagnostic performance. In CIN2, diagnostic accuracy improved relative to CIN1. Cytology achieved a sensitivity of 46.7% (95% CI, 21.3–73.4). CINtec^®^ outperformed cytology, with higher sensitivity (73.3%; 95% CI, 44.9–92.2) and comparable specificity (56.6%). PD-L1 showed very low sensitivity (6.7%; 95% CI, 0.2–31.9). Negative predictive values were highest for CINtec^®^ (93.3%) and cytology (89.9%), highlighting their relative reliability for ruling out CIN2.

Among CIN3 lesions, test performance increased substantially. Cytology showed a sensitivity of 72.7% (95% CI, 49.8–89.3). CINtec^®^ demonstrated high sensitivity at 100% (95% CI, 84.6–100), though with moderate specificity (65.2%). PD-L1 exhibited balanced performance, with a sensitivity of 63.6% and good specificity of 87.0%. Both CINtec^®^ and PD-L1 showed high NPVs (100% and 90.9%, respectively).

When considering CIN2+ lesions collectively, diagnostic accuracy improved further. Cytology reached a sensitivity of 71.4% (95% CI, 56.7–83.4) paired with a specificity of 100% (95% CI, 94.5–100.0), leading to a PPV of 100%. CINtec^®^ demonstrated the highest sensitivity (91.8%; 95% CI, 80.4–97.7), with high PPV (83.3%) and NPV (93.3%). PD-L1 showed lower sensitivity (46.9%) but high specificity (95.4%) and a PPV of 88.5%.

For CIN3+ lesions, cytology and CINtec^®^ again performed good. Cytology yielded a sensitivity of 82.4% (95% CI, 65.5–93.2). CINtec^®^ reached 100% sensitivity (95% CI, 89.7–100.0) with moderate specificity (75.0%). PD-L1 demonstrated moderate sensitivity (64.7%) but high specificity (95.0%), and a PPV of 84.6%.

For invasive carcinoma, cytology and CINtec^®^ showed high sensitivity (100%; 95% CI, 73.5–100). Cytology demonstrated higher specificity (77.5%) compared with CINtec^®^ (specificity 58.8%;). PD-L1 sensitivity was lower (66.7%; 95% CI, 34.9–90.1), but specificity was high (82.4%). Both cytology and CINtec^®^ achieved an NPV of 100%.

Given the referral-based nature of the study population, diagnostic performance estimates should be interpreted with caution. The observed high specificity and PPV for cytology in detecting CIN2+ should not be interpreted as true test performance in population-based screening settings.

Across lesion severities, cytology, CINtec^®^, and PD-L1 demonstrated markedly different discriminative abilities as measured by the area under the ROC curve (AUC) (Table 3).

For CIN2, cytology showed moderate accuracy (AUC, 0.681; 95% CI, 0.575–0.787), outperforming CINtec^®^ (0.650) and clearly exceeding PD-L1, which exhibited poor ability to distinguish CIN2 from non-CIN2 lesions (0.407). Performance improved substantially in CIN3, where both cytology (AUC, 0.820) and CINtec^®^ (0.826) showed high discriminative power, while PD-L1 also reached good accuracy (0.753).

In invasive carcinoma, cytology again demonstrated the highest AUC (0.887; 95% CI, 0.847–0.928), followed by CINtec^®^ (0.794) and PD-L1 (0.745). When lesions were grouped as CIN2+ and CIN3+, cytology achieved high discrimination (CIN2+ AUC, 0.937; CIN3+ AUC, 0.913), performing consistently better than CINtec^®^ (0.890 and 0.875), while PD-L1 showed only moderate diagnostic accuracy in both categories (0.712 and 0.799).

Overall, the results indicate that cytology provides the strongest ROC-based discriminative performance for higher-grade lesions (CIN2+, CIN3+, and invasive carcinoma), while CINtec^®^ performs comparably in CIN3 and CIN3+ but is less accurate for lower-grade lesions. PD-L1 shows generally limited discriminative ability, with modest improvement only in CIN3 and higher-grade categories.

**Figure 1:**
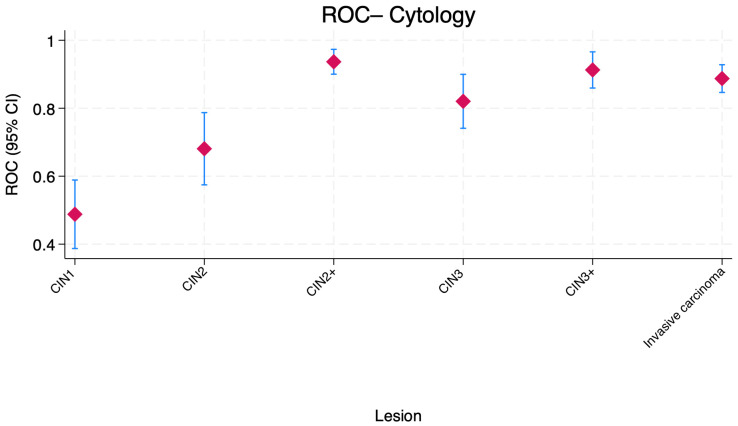
Boxplot comparison of ROC area under the curve (AUC) values for cytology combined with HR-HPV positivity across cervical lesion categories. Central markers represent the estimated AUC, while error bars indicate the corresponding 95% confidence intervals (CI). CIN—Cervical intraepithelial neoplasia.

Figure 1 shows the distribution of the AUC values for cytology combined with HR-HPV positivity across cervical lesion categories. For CIN1, the AUC was low and close to chance discrimination, consistent with weak performance in low-grade lesions. Accuracy increased substantially for CIN2 and CIN3, with AUC values clustering around 0.70–0.82. Notably, the highest AUC values were observed for CIN2+, CIN3+, and invasive carcinoma, each approaching or exceeding 0.90, indicating high discriminative ability in higher-grade lesions and invasive disease.

**Figure 2:**
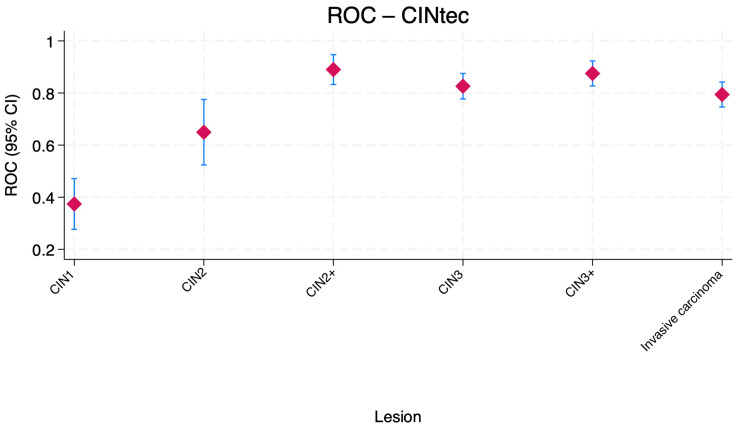
Boxplot comparison of ROC area under the curve (AUC) values for CINtec^®^ combined with HR-HPV positivity across cervical lesion categories. Central markers represent the estimated AUC, while error bars indicate the corresponding 95% confidence intervals (CI). CIN—Cervical intraepithelial neoplasia.

Figure 2 shows the distribution of the AUC values for CINtec^®^ combined with HR-HPV positivity across cervical lesion categories. The combination performed poorly for CIN1, with AUC values below 0.50, indicating weak discrimination. Performance improved for CIN2, with AUC values in the moderate range. The combination exhibited high accuracy for CIN3, where AUC values exceeded 0.80, and remained strong for CIN2+ and CIN3+, with values approaching 0.90. For invasive carcinoma, the AUC was slightly lower but remained in a favorable range (approximately 0.79).

**Figure 3:**
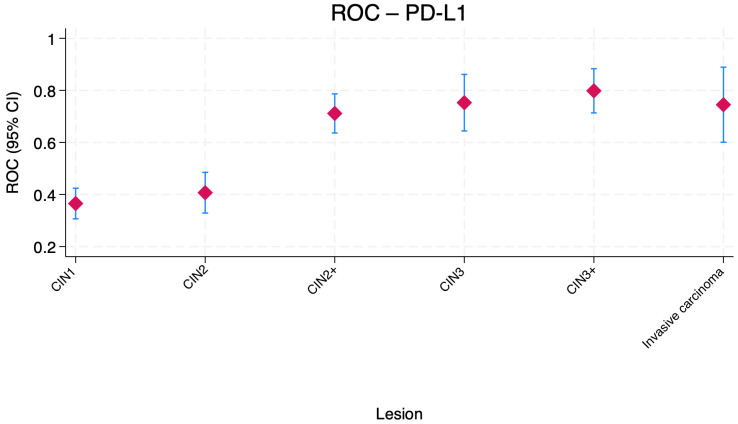
Boxplot comparison of ROC area under the curve (AUC) values for PD-L1 combined with HR-HPV positivity across cervical lesion categories. Central markers represent the estimated AUC, while error bars indicate the corresponding 95% confidence intervals (CI). CIN—Cervical intraepithelial neoplasia.

Figure 3 shows the distribution of the AUC values for PD-L1 ROC combined with HR-HPV positivity across cervical lesion categories. The AUC values for CIN1 and CIN2 were low, indicating poor ability to distinguish these lesions from non-lesions. Performance improved modestly for CIN2+ and CIN3+, reaching the 0.70–0.80 range, suggesting moderate accuracy for high-grade lesions. For invasive carcinoma, the combination maintained a similar moderate AUC around 0.75.

The discriminative performance of the combined tests (cytology, CINtec^®^, and PD-L1) increased progressively with lesion severity (Table 4). For CIN1, the ROC was 0.8222 (95% CI, 0.7248–0.9197), indicating good diagnostic accuracy. Accuracy improved further for CIN2, with an ROC of 0.9101 (95% CI, 0.8578–0.9623), and CIN3 (ROC = 0.9281, 95% CI, 0.8767–0.9795). The highest accuracy was observed in invasive carcinoma, for which the ROC reached 1, but this result is internally valid only within a high-prevalence cohort.

Cytology was the strongest predictor for low-grade lesions, particularly CIN1, where the coefficient was 2.127. In contrast, CINtec^®^ and PD-L1 demonstrated increasing predictive power for high-grade lesions. For CIN3, the coefficients were 1.261 for CINtec^®^ and 1.318 for PD-L1, and for invasive carcinoma, they were 0.865 and 0.752, respectively (Table 4).

Across all lesion categories, multivariable models that combined each test with high-risk HPV status demonstrated varying diagnostic performance (Table 5). Model 1 (Cytology + HR-HPV) showed consistently strong discrimination, with AUC values improving with lesion severity. The model performed moderately for CIN1 (AUC, 0.6902; 95% CI, 0.5851–0.7954) and achieved high accuracy for CIN2 (0.8701) and CIN3 (0.8873). For carcinoma, Model 1 reached an AUC of 1.

Model 2 (CINtec^®^ + HR-HPV) demonstrated weaker performance than Model 1 for CIN1 (AUC, 0.5343; 95% CI, 0.4224–0.6463), reflecting poor discriminatory ability at low-grade lesions. Accuracy improved for CIN2 (0.8261) and CIN3 (0.7941), but remained inferior to Model 1. Model 2 performed poorly for carcinoma (AUC, 0.5871), indicating limited utility for detecting invasive disease when combined with HR-HPV.

Model 3 (PD-L1 + HR-HPV) showed moderate accuracy for CIN1 (AUC, 0.7061; 95% CI, 0.6336–0.7785), outperforming Model 2 in this category and slightly exceeding Model 1. Discriminatory ability for CIN2 (0.7945) and CIN3 (0.7794) was moderate and lower than that of Model 1. For carcinoma, the model had an AUC of 0.7037, suggesting modest accuracy.

## 4. Discussion

In this prospective diagnostic accuracy study, we evaluated the performance of cervical cytology, CINtec^®^ (p16/Ki-67 dual immunostaining), and PD-L1 immunohistochemistry, individually and in combination with high-risk HPV (HR-HPV) testing, for the detection of cervical lesions confirmed by histopathology. The study also examined clinical and demographic characteristics associated with lesion severity. Importantly, all findings must be interpreted in the context of a referral-based cohort with a high prevalence of cervical disease, rather than a population-based screening setting.

Our results showed that for CIN1, all three tests demonstrated poor diagnostic performance. These findings align with existing evidence showing that screening tools designed primarily for high-grade lesions have limited relevance for CIN1, a category associated with high rates of spontaneous regression and limited clinical consequence. Therefore, in this study detection of CIN1 was not considered a clinically meaningful diagnostic target. Moreover, histology has limitations in this setting, including sampling error and interobserver variability, which reduce its reliability for low-grade disease and likely contributed to the poor test performance. The low sensitivity of CINtec^®^ for CIN1 is consistent with its biological target, as the assay detects cell cycle deregulation associated with transforming HPV infections rather than transient viral changes.

Importantly, these findings must be interpreted within the context of a referral-based study population. Such selection bias may exaggerate sensitivity estimates while limiting specificity, particularly for highly sensitive biomarkers. Taken together, the poor performance observed for CIN1 reinforces the limited clinical utility of detecting low-grade lesions and supports a risk-based diagnostic approach focused on identifying transforming infections and high-grade disease.

Diagnostic performance improved for CIN2, although it remained moderate overall. Cytology achieved a sensitivity of 46.7% and a specificity of 71.7%, reflecting intermediate accuracy. CINtec^®^ demonstrated higher sensitivity (73.3%) alongside lower specificity (56.6%), indicating effective detection at the cost of increased false positivity. PD-L1, with a sensitivity of 6.7% and an AUC of 0.407, offered negligible diagnostic value. Collectively, the results suggest that CINtec^®^ is more effective than cytology for identifying CIN2, though with reduced specificity, highlighting the trade-off between maximizing detection and limiting over-referral. However, the observed specificity estimates, particularly for cytology, must be interpreted cautiously, as the referral-based nature of the cohort likely reduces the number of true negative cases, leading to overestimation of specificity and positive predictive values.

For CIN3, all tests displayed markedly improved performance. Cytology yielded a sensitivity of 72.7% and a specificity of 79.3%, demonstrating strong clinical utility for high-grade lesions. CINtec^®^ reached a sensitivity of 100% and a specificity of 65.2%, with an NPV of 100%, indicating a high ability to exclude disease. PD-L1 showed a balanced profile, with a sensitivity of 63.6% and a specificity of 87.0%. The AUC values indicated that CINtec^®^ provided the highest discriminative capacity for CIN3 (AUC: 0.826). These findings reinforce the value of p16/Ki-67 dual staining as a powerful marker of transforming HPV infection.

For the composite endpoint CIN2+, cytology exhibited high specificity and PPV, but lower sensitivity (71.4%) than the other two tests. CINtec^®^ demonstrated high sensitivity (91.8%) and good specificity (86.2%), offering the most balanced diagnostic profile. PD-L1 maintained high specificity (95.4%) but low sensitivity (46.9%). The AUC values confirmed the high performance of CINtec^®^ in identifying CIN2+ lesions (AUC: 0.890). Nevertheless, the apparent strength of cytology, most notably the observation of high specificity for CIN2+, is unlikely to reflect real-world screening performance. In population-based screening settings, cytology specificity is consistently lower, and the absence of false positives observed here is best explained by spectrum and verification bias rather than true diagnostic accuracy.

For CIN3+, cytology demonstrated high sensitivity (82.4%) and specificity (91.2%), with an AUC value of 0.913. CINtec^®^ reached maximal sensitivity with moderate specificity (75.0%), while PD-L1 combined high specificity (95.0%) with moderate sensitivity (64.7%). The AUC values indicated that CINtec^®^ achieved the superior discriminative ability for CIN3+.

For invasive carcinoma, cytology and CINtec^®^ both achieved maximal sensitivity, yet cytology demonstrated superior specificity (77.5%) compared to CINtec^®^ (58.8%). PD-L1 showed high specificity (82.4%) with lower sensitivity (66.7%). AUC values confirmed cytology as the most accurate test for invasive disease detection within this population (AUC: 887). The present findings do not support the use of PD-L1 as a screening or triage biomarker for cervical intraepithelial neoplasia. Its low sensitivity and limited discriminatory capacity for CIN1–CIN2, and only moderate performance for CIN3+, argue against incorporation into screening algorithms. Instead, PD-L1 expression appears more consistent with a marker of immune escape and late-stage biological progression rather than early lesion detection.

The combined testing approach (cytology, CINtec^®^, and PD-L1) showed progressively stronger discriminative performance as lesion severity increased. While CIN1 demonstrated good accuracy (ROC 0.8222), performance improved notably for CIN2 (ROC 0.9101) and CIN3 (ROC 0.9281). The highest accuracy was observed for invasive carcinoma. These findings indicate that multimodal testing provides a substantial diagnostic advantage, particularly for high-grade lesions and invasive disease.

When incorporated into multivariable models, cytology combined with HR-HPV consistently demonstrated the strongest diagnostic performance across all lesion categories, achieving high discrimination for invasive carcinoma. CINtec^®^ + HR-HPV and PD-L1 + HR-HPV showed moderate accuracy for CIN2 and CIN3 but performed poorly for CIN1 and carcinoma, offering limited added value compared with cytology-based modeling.

While these findings suggest complementary roles for biomarker-based triage in high-risk settings, they should not be interpreted as providing population-level estimates of screening performance. Comparisons with published literature further highlight this limitation. Large population-based screening studies and colposcopy-referred cohorts consistently report lower specificity for cytology and smaller performance differentials between cytology and CINtec^®^ than those observed in the present study. Accordingly, although our findings are directionally consistent with prior evidence supporting the role of p16/Ki-67 dual staining in the identification of transforming HPV infections, the absolute diagnostic performance estimates reported here are likely to overestimate accuracy in routine clinical screening settings.

Literature data showed that for CIN1, cytology demonstrated high sensitivity, but moderate specificity, and its ability to distinguish CIN1 from higher or lower grades is limited [16]. Also, for CIN1 or higher, p16/Ki-67 dual staining showed high sensitivity (up to 89%) and moderate specificity (73.5%) [17]. However, its ability to specifically identify CIN1 (as opposed to CIN2+) is limited, as p16/Ki-67 positivity increases with lesion severity and is much stronger in CIN2/3 than in CIN1 [18,19]. In CIN1, PD-L1 expression is higher than in normal tissue but lower than in high-grade lesions or cancer [12,20].

Moreover, for the detection of CIN2+ lesions, cytology demonstrated a sensitivity ranging from 62% and 78% [2,21,22,23]. For CIN3+ (grade 3 or worse), cytology sensitivity varied from 70 to 94% [23,24].

CINtec^®^ test showed higher sensitivity and specificity for high-grade lesions. For CIN2+, sensitivity ranged from 83% to 95% and specificity from 58% to 89% [2,25,26,27,28,29]. For CIN3+, sensitivity was between 86% and 94%, and specificity ranges from 51% to 79% [2,25,26,27,28,29]. PD-L1 expression is not robustly reported for CIN2+ or CIN3+ detection in the context of primary screening. While PD-L1 is highly expressed in invasive cancer and may have prognostic value, its diagnostic utility for CIN2/3 is limited and not well established in the literature [12,20,30].

In a cross-sectional study of women aged 45 years and older with transformation zone type 3, Gustafson et al., reported that CINtec^®^ achieved a sensitivity of 96.7% and a negative predictive value of 97.6% for the detection of CIN2+, significantly outperforming cytology, which showed a sensitivity of 70.0% and an NPV of 86.4% [31]. In contrast, cytology demonstrated superior specificity (90.5% vs. 63.5%) and positive predictive value (77.8% vs. 55.8%) compared with CINtec^®^ [31]. Further evidence supporting the diagnostic role of CINtec^®^ comes from the STAIN-IT study by El-Zein et al., which evaluated its performance in a large colposcopy-referred population [32]. In this study of 492 cervical specimens with histological verification, CINtec^®^ achieved a sensitivity of 80.7% and a specificity of 64.0% for the detection of CIN2+, while for CIN3+ sensitivity increased to 86.8% with a specificity of 54.0%. Compared with HPV testing, CINtec^®^ showed slightly lower sensitivity but consistently higher specificity, improving specificity by 7.9% for CIN2+ and 9.6% for CIN3+ [32].

Recent work by Huang et al., further supports the diagnostic value of cell-cycle–based biomarkers for identifying clinically significant cervical disease [33]. In a cohort of 403 women undergoing cervical evaluation, p16 (the key component of CINtec^®^) demonstrated strong performance for CIN2+ detection, with a sensitivity of 82.2% and a specificity of 90.1%, outperforming both E6/E7 mRNA testing (sensitivity 68.2%, specificity 61.8%) and Ki-67 alone. While Ki-67 showed very high sensitivity (95%), its poor specificity (27.2%) limited its clinical utility as an isolated marker. Importantly, the combined p16/Ki-67 approach (CINtec^®^) achieved the most balanced diagnostic profile, reaching 90.0% sensitivity and 79.8% specificity for CIN2+ lesions, significantly improving accuracy compared with single-marker strategies.

Although the literature data do not report diagnostic performance metrics for PD-L1, a systematic review found that PD-L1 expression increases from LSIL (CIN1) through HSIL (CIN2/3) to invasive cancer, in both epithelial and mononuclear cells, supporting a role in the progression and/or persistence of CIN2+ and cancer [30].

### 4.1. Limitations

The results of this study should be interpreted considering the following limitations: its single center design, small sample of patients, and limited diagnostic subgroups. PD-L1 assessment represented another important limitation of this study, as cutoffs, antibody clones, and scoring systems were not standardized or clinically validated for cervical intraepithelial neoplasia. Although a ≥1% TPS cutoff was based on prior exploratory studies, it remains arbitrary. The CPS was not applied because it was not validated for cervical dysplasia, and this aspect may limit the assessment of the immune microenvironment as well as contribute to the modest performance of PD-L1.

Moreover, the study included patients who were referred for screening and evaluation to a tertiary medical unit, which might contribute to the high prevalence of high-grade cervical dysplasia in the evaluated cohort.

### 4.2. Clinical Implications

The combined model of cytology and high-risk HPV status demonstrated the most consistent diagnostic performance across clinically relevant endpoints, particularly for CIN2+, CIN3, and carcinoma. This finding supports the continued use of cytology-based evaluation in HPV-positive patients, in line with current screening algorithms.

On the other hand, the combination of CINtec^®^ with HR-HPV did not improve diagnostic performance relative to cytology + HR-HPV and showed inferior discrimination for carcinoma, limiting its clinical utility as a standalone testing strategy in this cohort of patients.

Also, the PD-L1 + HR-HPV model did not outperform cytology + HR-HPV for the diagnosis of CIN2+, CIN3, or carcinoma. Thus, PD-L1 did not provide incremental value when incorporated into diagnostic strategies. Overall, these findings argue against the inclusion of PD-L1 in multivariable triage models for cervical disease and support its interpretation as a marker of biological progression rather than a clinically useful diagnostic tool.

The findings support the potential role of CINtec^®^ as a triage tool in high-risk or referral settings. However, given the high-prevalence cohort and resulting spectrum bias, these results should not be used to infer screening performance.

Subsequent research should include larger, multi-center cohorts and longitudinal follow-up to assess the temporal predictive significance of cytology, CINtec^®^, and PD-L1, especially for HPV persistence and lesion progression.

## 5. Conclusions

This prospective diagnostic accuracy study has shown that the efficacy of the assessed tests was significantly influenced by lesion severity. Cytology, CINtec^®^, and PD-L1 exhibited inadequate diagnostic accuracy for CIN1, indicating that low-grade lesions were not consistently identified using these tests.

Both cytology and CINtec^®^ have shown significantly enhanced performance for CIN2 and particularly CIN3 detection, with CINtec^®^ attaining the best sensitivity and the highest AUC values, alongside the highest negative predictive values.

Cytology, although less sensitive than CINtec^®^ for high-grade precancerous lesions, demonstrated high accuracy for CIN2+ and CIN3+ and proved to be the most effective single test for invasive carcinoma, exhibiting the highest AUC and superior specificity relative to CINtec^®^ and PD-L1.

As far as we know, this is the first study in the literature that reports the diagnostic performance metrics for PD-L1 for cervical dysplasia and cervical carcinoma. PD-L1 immunohistochemistry demonstrated limited diagnostic efficacy as a primary assay, exhibiting poor sensitivity and only moderate AUC values across most lesion categories, while maintaining reasonably high specificity for high-grade lesions and invasive cancer. Our results do not support the inclusion of PD-L1 in multivariable triage models for cervical disease and support its interpretation as a marker of biological progression rather than a clinically useful diagnostic or screening tool.

The integration of combined testing (cytology, CINtec^®^, and PD-L1) with multivariable models that incorporate high-risk HPV status can enhance lesion detection, especially for CIN2+, CIN3+, and invasive cancer.

Because this study was conducted in a high-prevalence, referral-based cohort, diagnostic performance, particularly specificity, may be overestimated and should not be interpreted as representative of population-based screening.

## Figures and Tables

**Table 1:** ANOVA analysis of continuous variables between groups.

Variable	Biopsy Group	Mean	SD	ANOVA *p*-Value
Age (years)	CIN1	34.65	9.37	0.0031
	CIN2	37.07	9.42	
	CIN3	36.14	8.75	
	Invasive carcinoma	44.58	8.06	
	Negative	41.88	9.67	
BMI (kg/m^2^)	CIN1	25.18	3.79	0.0218
	CIN2	23.29	3.76	
	CIN3	22.84	4.11	
	Invasive carcinoma	27.24	3.72	
	Negative	24.69	4.16	
Number of pregnancies	CIN1	0.90	1.01	0.167
	CIN2	0.64	0.74	
	CIN3	1.09	0.92	
	Invasive carcinoma	1.58	1.56	
	Negative	0.88	0.91	

Legend: CIN—cervical intraepithelial neoplasia; BMI—body mass index, SD—standard deviation.

**Table 2:** Baseline clinical and demographic characteristics according to histopathologic diagnosis.

Variable	CIN1 n (%)	CIN2 n (%)	CIN3 n (%)	Invasive Carcinoma n (%)	Negative n (%)	*p*-Value
Residence (Rural)	10 (33.3%)	4 (13.3%)	7 (23.3%)	4 (13.3%)	5 (16.7%)	0.466
Residence (Urban)	21 (25.0%)	11 (13.1%)	15 (17.9%)	8 (9.5%)	29 (34.5%)	
History of HPV infection	31 (34.1%) ***	14 (15.4%) **	21 (23.1%) ***	11 (12.1%) **	14 (15.4%)	<0.0001
History of STIs	0 (0%)	3 (75%) **	0 (0%)	0 (0%)	1 (25%)	0.006
Smoking history	5 (16.7%)	7 (23.3%) **	10 (33.3%) **	5 (16.7%) *	3 (10%)	0.012
Alcohol consumption	0 (0%)	2 (18.2%)	2 (18.2%)	3 (27.3%)	4 (36.4%)	0.137
Number of sexual partners	30 (96.8%)	15 (100%)	22 (100%)	12 (100%)	33 (100%)	0.955
Hormonal contraceptive use	3 (9.7%)	4 (12.9%)	8 (25.8%)	5 (16.1%)	11 (35.5%)	0.113
Immunosuppression	1 (50%)	0 (0%)	0 (0%)	0 (0%)	1 (50%)	0.819
HPV vaccination	13 (40.6%)	3 (9.4%)	9 (28.1%)	0 (0%)	7 (21.9%)	0.028
High-risk HPV positivity	28 (30.1%) ***	15 (16.1%) ***	22 (23.7%) ***	12 (12.9%) *	16 (17.2%)	<0.0001
Low-risk HPV positivity	6 (100%) **	0 (0%)	0 (0%)	0 (0%)	0 (0%)	0.002
Coinfection	16 (38.1%)	6 (14.3%)	7 (16.7%)	6 (14.3%)	7 (16.7%)	0.093
ASC-H	0 (0%)	0 (0%)	2 (100%)	0 (0%)	0 (0%)	<0.0001
ASCUS	0 (0%)	0 (0%)	1 (100%)	0 (0%)	0 (0%)	
HSIL	0 (0%)	7 (20.0%)	16 (45.7%) **	12 (34.3%) **	0 (0%)	
LSIL	31 (73.8%) ***	8 (19.0%)	3 (7.1%)	0 (0%)	0 (0%)	
NILM	0 (0%)	0 (0%)	0 (0%)	0 (0%)	34 (100%)	
CINtec^®^ positivity	9 (16.7%) **	11 (20.4%) ***	22 (40.7%) ***	12 (22.2%) ***	0 (0%)	<0.0001
PD-L1 positivity	1 (3.9%)	1 (3.9%)	14 (53.9%) ***	8 (30.8%) ***	2 (7.7%)	<0.0001

Legend: CIN—Cervical intraepithelial neoplasia; HPV—Human papillomavirus; STIs—Sexually transmitted infections; ASC-H—Atypical squamous cells, cannot exclude HSIL; ASCUS—Atypical squamous cells of undetermined significance; HSIL—High-grade squamous intraepithelial lesion; LSIL—Low-grade squamous intraepithelial lesion; NILM—Negative for intraepithelial lesion or malignancy; CINtec^®^—p16/Ki-67 dual immunostaining test; PD-L1—Programmed death-ligand 1. Post hoc pairwise comparisons were performed between each biopsy category and the Negative group with Bonferroni correction. * *p* < 0.05, ** *p* < 0.01, *** *p* < 0.001 indicate statistically significant differences compared with the negative group.

**Table 3:** Diagnostic performance of cytology, CINtec^®^, and PD-L1 for the detection of cervical lesions across histopathologic categories.

Type of Lesion	Test	Sensitivity (95% CI)	Specificity (95% CI)	PPV (95% CI)	NPV (95% CI)	ROC (95% CI)
CIN1	Cytology	0.0% (0–11.2)	57.8% (46.5–68.6)	0.0% (0–10.0)	60.8% (49.1–71.6)	0.488 (0.387–0.589)
	CINtec^®^	29.0% (14.2–48.0)	45.8% (34.8–57.1)	16.7% (7.9–29.3)	63.3% (49.9–75.4)	0.374 (0.277–0.472)
	PD-L1	3.2% (0.1–16.7)	69.9% (58.8–79.5)	3.8% (0.1–19.6)	65.9% (55.0–75.7)	0.366 (0.307–0.424)
CIN2	Cytology	46.7% (21.3–73.4)	71.7% (61.8–80.3)	20.0% (8.4–36.9)	89.9% (81.0–95.5)	0.681 (0.575–0.787)
	CINtec^®^	73.3% (44.9–92.2)	56.6% (46.2–66.5)	20.4% (10.6–33.5)	93.3% (83.8–98.2)	0.650 (0.524–0.775)
	PD-L1	6.7% (0.2–31.9)	74.7% (65.0–82.9)	3.8% (0.1–19.6)	84.1% (74.8–91.0)	0.407 (0.329–0.485)
CIN3	Cytology	72.7% (49.8–89.3)	79.3% (69.6–87.1)	45.7% (28.8–63.4)	92.4% (84.2–97.2)	0.820 (0.741–0.900)
	CINtec^®^	100% (84.6–100)	65.2% (54.6–74.9)	40.7% (27.6–55.0)	100% (94–100)	0.826 (0.777–0.875)
	PD-L1	63.6% (40.7–82.8)	87.0% (78.3–93.1)	53.8% (33.4–73.4)	90.9% (82.9–96.0)	0.753 (0.644–0.862)
CIN2+	Cytology	71.4 (56.7–83.4)	100.0 (94.5–100.0)	100.0 (90.0–100.0)	82.3 (72.1–90.0)	0.937 (0.900–0.973)
	CINtec^®^	91.8 (80.4–97.7)	86.2 (75.3–93.5)	83.3 (70.7–92.1)	93.3 (83.8–98.2)	0.890 (0.833–0.947)
	PD-L1	46.9 (32.5–61.7)	95.4 (87.1–99.0)	88.5 (69.8–97.6)	70.5 (59.8–79.7)	0.712 (0.636–0.787)
CIN3+	Cytology	82.4 (65.5–93.2)	91.2 (82.8–96.4)	80.0 (63.1–91.6)	92.4 (84.2–97.2)	0.913 (0.859–0.966)
	CINtec^®^	100.0 (89.7–100.0)	75.0 (64.1–84.0)	63.0 (48.7–75.7)	100.0 (94.0–100.0)	0.875 (0.827–0.923)
	PD-L1	64.7 (46.5–80.3)	95.0 (87.7–98.6)	84.6 (65.1–95.6)	86.4 (77.4–92.8)	0.799 (0.714–0.884)
Invasive carcinoma	Cytology	100% (73.5–100)	77.5% (68.1–85.1)	34.3% (19.1–52.2)	100% (95.4–100)	0.887 (0.847–0.928)
	CINtec^®^	100% (73.5–100)	58.8% (48.6–68.5)	22.2% (12.0–35.6)	100% (94–100)	0.794 (0.746–0.842)
	PD-L1	66.7% (34.9–90.1)	82.4% (73.6–89.2)	30.8% (14.3–51.8)	95.5% (88.8–98.7)	0.745 (0.601–0.889)

Legend: CIN—cervical intraepithelial neoplasia; CINtec^®^—p16/Ki-67 dual immunostaining; PD-L1—programmed death-ligand 1; PPV—positive predictive value; NPV—negative predictive value; ROC— receiver-operating-characteristic curve.

**Table 4:** Receiver-operating-characteristic performance of combined tests across cervical lesion categories along with β coefficients derived from the multivariable logistic regression model.

Biopsy Category	ROC	Std. Error	95% CI	Cytology (β)	CINtec^®^ (β)	PD-L1 (β)
CIN1	0.8222	0.0497	0.7248–0.9197	2.1270	–1.4695	–1.1827
CIN2	0.9101	0.0267	0.8578–0.9623	0.3297	0.8402	–1.0510
CIN3	0.9281	0.0262	0.8767–0.9795	–0.1228	1.2612	1.3180
Invasive carcinoma	1.0000	0.0000	1.0000–1.0000	–0.3432	0.8650	0.7521

Legend: CIN—cervical intraepithelial neoplasia; ROC—receiver-operating-characteristic curve; CI—confidence interval; PD-L1—programmed death-ligand 1.

**Table 5:** Diagnostic performance of HPV genotyping and screening tests for the detection of cervical lesions across histopathologic categories.

Model	Predictors Included	Lesion	AUC	Std. Error	95% CI
Model 1	Cytology + HR-HPV	CIN1	0.6902	0.0537	0.5851–0.7954
		CIN2	0.8701	0.0319	0.8075–0.9326
		CIN3	0.8873	0.0208	0.8465–0.9280
		Carcinoma	1.0000	0.0000	1.0000–1.0000
Model 2	CINtec^®^ + HR-HPV	CIN1	0.5343	0.0571	0.4224–0.6463
		CIN2	0.8261	0.0250	0.7772–0.8750
		CIN3	0.7941	0.0245	0.7461–0.8421
		Carcinoma	0.5871	0.0741	0.4419–0.7323
Model 3	PD-L1 + HR-HPV	CIN1	0.7061	0.0369	0.6336–0.7785
		CIN2	0.7945	0.0456	0.7052–0.8838
		CIN3	0.7794	0.0604	0.6610–0.8978
		Carcinoma	0.7037	0.0568	0.5923–0.8150

Legend: CIN—cervical intraepithelial neoplasia; CINtec^®^—p16/Ki-67 dual immunostaining; PD-L1—programmed death-ligand 1; AUC—area under the ROC curve; CI—confidence interval; HR-HPV—high-risk human papillomavirus.

## Data Availability

The original contributions presented in this study are included in the article/Supplementary Material. Further inquiries can be directed to the corresponding author.

## Supplementary Materials

The following supporting information can be downloaded at: https://www.mdpi.com/article/10.3390/jcm15031171/s1, Table S1: Age comparison between groups (Bonferroni); Table S2: BMI comparison between groups (Bonferroni); Table S3: Number of pregnancy comparisons between groups (Bonferroni).

## Abbreviations

The following abbreviations are used in this manuscript:

CIN	Cervical intraepithelial neoplasia
CIN2+	CIN2, CIN3, or invasive carcinoma
CIN3+	CIN3 or invasive carcinoma
CINtec^®^	p16/Ki-67 dual immunostaining test
PD-L1	Programmed death-ligand 1
HPV	Human papillomavirus
HR-HPV	High-risk human papillomavirus
LR-HPV	Low-risk human papillomavirus
ROC	Receiver-operating-characteristic
AUC	Area under the ROC curve
PPV	Positive predictive value
NPV	Negative predictive value
LSIL	Low-grade squamous intraepithelial lesion
HSIL	High-grade squamous intraepithelial lesion
ASC-US	Atypical squamous cells of undetermined significance
ASC-H	Atypical squamous cells, cannot exclude HSIL
NILM	Negative for intraepithelial lesion or malignancy
BMI	Body mass index
SD	Standard deviation
CI	Confidence interval
