# Supplementary material for: Prospective Evaluation of Cytology, CINtec® and PD-L1 for the Detection of Cervical Intraepithelial Neoplasia: A Single-Center Study
Source: J Clin Med. 2026 Feb 2;15(3):1171. doi: 10.3390/jcm15031171 (PMC12897607; doi:10.3390/jcm15031171)
Supplement: Supplementary file 1 [file jcm-15-01171-s001.zip › Table S3.pdf]

Table S3: Number of pregnancy comparisons between groups (Bonferroni)

**Row Mean / Col Mean**	**CIN1**	**CIN2**	**CIN3**	**Carcinoma**
**CIN2**	-0.260369	—	—	—
p-value	1.000	—	—	—
**CIN3**	0.187683	0.448052	—	—
p-value	1.000	1.000	—	—
**Carcinoma**	0.680108	0.940476	0.492424	—
p-value	0.502	0.197	1.000	—
**Negative**	-0.020873	0.239496	-0.208556	-0.70098
p-value	1.000	1.000	1.000	0.411
