# Supplementary material for: Prospective Evaluation of Cytology, CINtec® and PD-L1 for the Detection of Cervical Intraepithelial Neoplasia: A Single-Center Study
Source: J Clin Med. 2026 Feb 2;15(3):1171. doi: 10.3390/jcm15031171 (PMC12897607; doi:10.3390/jcm15031171)
Supplement: Supplementary file 1 [file jcm-15-01171-s001.zip › Table S1.pdf]

Table S1 – Age comparison between groups (Bonferroni)

**Row Mean / Col Mean**	**CIN1**	**CIN2**	**CIN3**	**Carcinoma**
**CIN2**	2.42151	—	—	—
p-value	1.000	—	—	—
**CIN3**	1.4912	-0.930303	—	—
p-value	1.000	1.000	—	—
**Carcinoma**	9.93817	7.51667	8.44697	—
p-value	0.020	0.378	0.122	—
**Negative**	7.23719	4.81569	5.74599	-2.70098
p-value	0.021	0.952	0.249	1.000
