# Supplementary material for: Prospective Evaluation of Cytology, CINtec® and PD-L1 for the Detection of Cervical Intraepithelial Neoplasia: A Single-Center Study
Source: J Clin Med. 2026 Feb 2;15(3):1171. doi: 10.3390/jcm15031171 (PMC12897607; doi:10.3390/jcm15031171)
Supplement: Supplementary file 1 [file jcm-15-01171-s001.zip › Table S2.pdf]

Table S2: BMI comparison between groups (Bonferroni)

**Row Mean / Col Mean**	**CIN1**	**CIN2**	**CIN3**	**Carcinoma**
**CIN2**	-1.89	—	—	—
p-value	1.000	—	—	—
**CIN3**	-2.34	-0.45	—	—
p-value	0.362	1.000	—	—
**Carcinoma**	2.05	3.95	4.39	—
p-value	1.000	0.114	0.025	—
**Negative**	-0.50	1.40	1.84	-2.55
p-value	1.000	1.000	0.915	0.575
